# General practitioners in front of COVID-19: Italy in European comparative perspective

**DOI:** 10.3389/fsoc.2024.1365517

**Published:** 2024-05-23

**Authors:** Angela Genova, Simone Lombardini

**Affiliations:** ^1^Department of Economics, Society, Politics, University of Urbino, Urbino, Italy; ^2^Department of Economics, University of Genoa, Genoa, Italy

**Keywords:** primary care, health care systems, death rate, health care performance, aging population, pandemic, mortality

## Abstract

COVID-19 has highlighted strengths and weaknesses in healthcare systems all over the word. Despite the differences in primary care models in Europe, this study investigates the state-of-the-art of general practitioners (GPs) before the COVID-19 pandemic spread as a result of the reform process of the previous two decades. The GPs numbers over 100,000 inhabitants has been considered as a proxy of public health investment in GPs. Is the number of GPs increased or decreased in the last 20 years of reform processes in European countries? The main hypothesis is that European healthcare systems would have increased the number of GPs coherently with WHO recommendations. Comparative data on the number of GPs per 100,000 inhabitants in 21 European countries are investigated between 1995 and 2014 (the last available data). Data show that the number of family doctors over 100,000 inhabitants in European countries has increased over the last 20 years, except for Italy, where it has strongly reduced. Primary care has had a crucial role in managing the pandemic. Results of this study suggest that a country such as Italy, which has not invested in family doctors in the last two decades, would have been less equipped to manage the COVID-19 pandemic.

## Introduction

The COVID-19 pandemic spread throughout European countries at the beginning of 2020, and among them, Italy had some of the highest incidence of COVID-19 deaths (Statista, 2020). The pandemic has spread differently among European countries, even if comparative analysis presents limited data (ECDC, European Centre for Disease Prevention and Control An agency of the European Union, 2020). Healthcare service workers at all levels—primary care, hospital and community care, and highly specialized treatment facilities—have been protagonists in managing the pandemic within the wide coronophobia spread (Arora et al., 2020; Asmundson and Taylor, 2020; Lee et al., 2020). COVID-19 has been an enormous challenge for all healthcare systems, bringing to light several national healthcare system strengths and weaknesses (Legido-Quigley et al., 2020).

Primary care services (Greenhalgh et al., 2020) have played a key part in managing the pandemic at national and local levels (Ares-Blanco et al., 2023; Khalil-Khan and Khan, 2023), with general practitioners (GPs) being a frontline emergency profession (Adams and Walls, 2020), playing a key role of public leadership (Suar et al., 2023). Despite the differences in their role and function in Europe (Grielen et al., 2000; Glonti et al., 2015; Erlend et al., 2017; Groenewegen et al., 2020), GP offer and organization are crucial aspects of healthcare systems in the context of the World Health Organization’s (WHO’s) policy frame (Gulliford, 2002; Rico et al., 2003).

The key role of primary care in healthcare systems, and therefore also of GPs, is even more crucial in the context of the aging population (Liotta, 2020). Moreover, GPs have been a paramount point of analysis for healthcare policy systems during the COVID-19 pandemic emergency, as confirmed by the message of the WONCA Executive Committee on World Family Doctor Day on 19 May 2020 (WONCA, 2020; OECD, 2021).

The importance of GPs in healthcare system organization is well-known (Gulliford, 2002). As patients’ first and main point of entry into the healthcare system, GPs play a strategic role in assessing health needs, as well as in coordinating with other health services (Kringos et al., 2010). They also affect healthcare efficiency, which is a key profession in primary care (Starfield et al., 2005; Haggerty et al., 2013): “Strong primary care is associated with better population health and lower rate of unnecessary hospitalizations” (Kringos et al., 2013), considering the key role of GPs as gatekeeper to the rest of healthcare services (Glonti et al., 2015). Assessing primary care organization and performance has been the focus of a recent study presenting a literature review synthesis and the proposal of a theoretical and practical framework (Senn et al., 2021). Structural aspects, such as the number of GPs, nurses, social workers, and pharmacists, are fundamental aspects of such a framework because they are likely to have a significant impact on the performance of the healthcare systems (Hogg et al., 2008).

This study is not going to investigate the performance of healthcare systems in Europe, but it is focusing just on a preliminary analysis of structural data on GPs in a European comparative perspective, with specific attention to the Italian case study. The GPs numbers over 100,000 inhabitants (considered together to the degree of an aging population) has been considered as a proxy of public health investment in family doctors. Is the number of GP family doctors per 100.000 in. increased or decreased in the last 20 years of reform processes in European countries? The main hypothesis is that European healthcare systems would have increased their investments in GPs, increasing their number, coherently with WHO recommendations.

In recent years, several studies have highlighted the critical flaws and weaknesses of the Italian healthcare system (Ferré et al., 2014; Petmesidou et al., 2020; Giarelli, 2021; Neri, 2021). Specific analyses have also investigated and discussed personnel healthcare policy (Vicarelli and Pavolini, 2015; Pavolini et al., 2018). Although several analyses have addressed the effect of the reform process on the Italian national healthcare system, just a few have specifically dealt with the GPs sector’s reform in Italy (Cipolla et al., 2006; Clemente et al., 2021; Genova et al., 2021).

GPs are the main protagonists of primary care in Italy due to the limited role of other health and social professionals, such as community nurses, pharmacists, and social workers, as highlighted in the recent primary care reforms passed in 2022 (Ingrosso, 2023; Mauro and Giancotti, 2023). Community/family nurses, in fact, have not yet been fully implemented in Italy (Del Vecchio et al., 2017), as well as specific healthcare services to manage the pandemic at the local level (USCA) (Corte dei Conti, 2020).

Due to the key role of GPs’ activities in the Italian context (Ferré et al., 2014), this study intends to fill this gap by investigating GPs’ structural data before the arrival of the pandemic in Italy from a European comparative perspective. Therefore, this study investigates the availability of GP services, in Italy, in terms of the number of GPs in the population, as the result of policy-reform processes in the last decades, in a European comparative perspective.

## Methods

### Data from a comparative perspective

This study provides a European comparative perspective of GP data by looking at the Health for All (HFA, European Health Information Gateway, 2022) database. The HFA database provides a dataset of GPs per 100,000 inhabitants for a large number of countries. The time series considered starts in 1995 and ends in 2014; unfortunately, the database did not gather data after this year. The limitations of this study are highly linked to the limitation of MMGs data; it is difficult to know why the HFA database has not been updated for 10 years. However, the comparison is made with several countries (22) and shows us the general trend in Europe and also the outlier countries that experienced an opposite trend of growth.^1^ Moreover, we compensate the HFA holes, deepening the particular case of Italy, using MMGs data provided by ISTAT, which are updated until 2021. After showing the GPs number of these European countries, the study compares it to the COVID-19 mortality rate among the same countries, looking at the Worldometers database (the cutoff is set at 12/31/2021). Finally, this study compares the Italian number of GPs to the European average.

## Results

### Italian GPs from a comparative European perspective

The main piece of data of this study from the European comparative perspective is the number of GPs per 100,000 inhabitants for the 21 available European countries in 1995, 2005, and 2014 (Table 1). Despite the different roles that GPs might have in different healthcare systems (Groenewegen et al., 2014), analysis shows an increasing number of GPs in almost all European countries. A great variation among countries reflects differences in health systems (Wendt et al., 2009). The lowest values were found for Greece and Poland, with around the same relative number in 2005 (approximately 14 general practitioners per 100,000 inhabitants); for France and Belgium, the highest values were far more than 100 general practitioners per 100,000 inhabitants. The standard deviation is 33.99 in 2005 and 29.15 in 2015. This means that the number of general practitioners among countries may vary between +/−30 around the mean. This is a huge value considering that the mean of general practitioners per 100,000 inhabitants among European countries was 68.48 in 2005 and 74.64 in 2015. These data are also associated (the last column of Table 1) with the percentage variation of people over 65 years to capture the evolution of the population structure over time.

**Table 1:** GPs per 100,000 people.

Country	1995	2005	2014	% variation	% variation people over 65 years
				GP	
Albania	60.11	52.9	55.86	−7.07	99.33
Austria	66.57	76.18	77.66	16.66	21.62
Belgium	–	118.38	111.88	−5.49	13.25
Bulgaria	–	67.6	62.84	−7.04	31.75
Croatia	–	–	57	–	36.05
Czech Rep.	69.45	72.53	–	–	33.16
Estonia		68.87	71.8	4.25	37.24
France	166.36	169.96	159.83	−3.93	21.45
Germany	66.38	66.61	66.87	0.74	35.44
Greece	–	14.31	39.15	173.58	32.73
Ireland	45.87	51.52	76.68	67.17	11.11
Italy	82.96	81.12	75.16	−9.4	29.31
Lithuania	38.47	67.24	89.14	131.71	50.58
Luxembourg	-	78.25	87.86	12.28	0.88
Netherlands	46.56	66.15	79.84	71.48	32.68
Poland	-	14.27	21.85	53.12	34.06
Portugal	40.21	46.48	58.99	46.7	34.52
Romania	–	–	–	–	39.65
Slovenia	–	38.18	51.5	34.89	43.54
Spain	–	71.92	75.4	4.84	20.94
Sweden	48.2	59.42	–	–	11.75
United Kingdom	–	72.31	80.02	10.66	14.34

Source: HFA.

The majority of European countries have increased the amount of GPs employed over the last 20 years. In France and Belgium, on the opposite, numbers slightly fell, but France remains the country with the highest rate of GPs employed (double in spite of the other countries). Among the other countries, the Netherlands exhibits the highest increment. Germany remained constant, and Greece had the lowest rate in Europe, but it constantly improved. Portugal exhibited a more constant trend of growth. Only Spain seems to be constant, but the gaps in the time series do not let us know its past trend.

Overall, the rate of GP growth relative to population has grown in the eastern countries over the last 20 years. A lot of them showed a period of stability close to a value of 70 between 2002 and 2010, then continued to increase. Among the others, Lithuania’s trend stands out, becoming one of the highest values in Europe and the highest absolute value for the eastern countries. The lowest one is Poland, both for 1995 and 2014, even if its value has improved (tripled).

Ireland experienced slight growth until 2010. Then, GP reached the level of 75 in just 5 years. A more stable growth trend was observed for Sweden. The United Kingdom improved its value, then remained constant.

**Figure 1:**
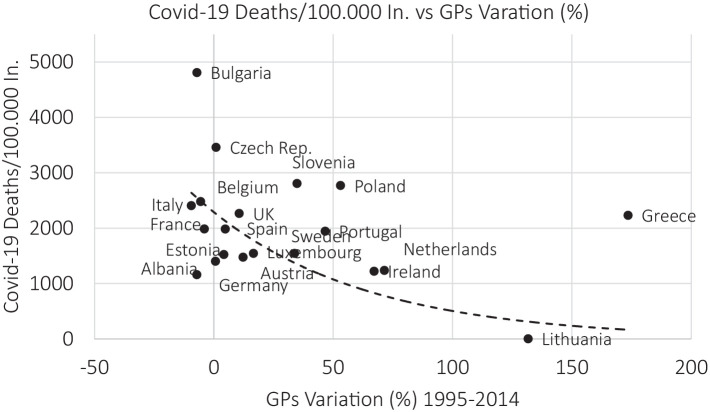
The percentage variation GPs per 100.000 in. vs. COVID-19 deaths. Source: HFA and Worldometers Database, authors’ elaborations.

The GPs over 100,000 in. rate could have affected the final outcome of the Public Health systems in Europe during the stress test represented by the COVID-19 pandemic. A suggestion of this hypothesis comes from a scatter plot that compares the percentage variation in GPs over 100,000 in. to the number of people dead of/with COVID-19 in 2020 over population. In Figure 1 we observe that there is a negative correlation (well approximated by an exponential function with negative exponent) between these two variables. Countries which have increased, in the past decades, their GPs number, have meanwhile experienced a lower COVID-19 mortality rate, and vice versa.

**Figure 2:**
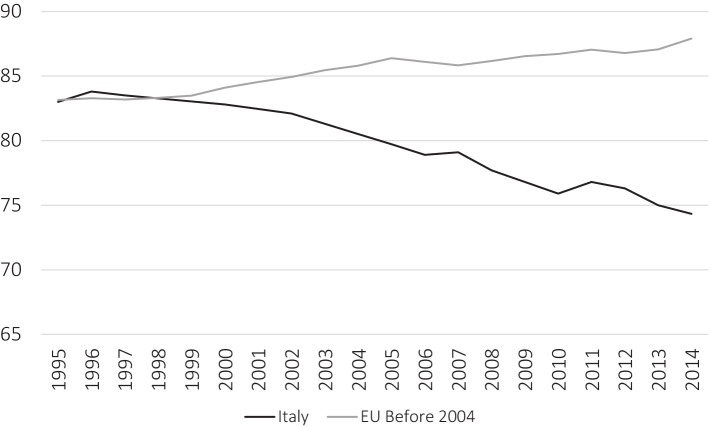
Trend in GP per 100,000 inhabitants in Italy and EU member state (before 2004) average. Source: HFA database, our elaboration (Permission required).

In contrast with the European trend, in Italy, we found a decreasing process (Figure 2): the number of GPs dropped from the second to the ninth in absolute value in Europe. The Italian case represents the worst one in Europe, in terms of growth trend. The loss of GPs has hit Italy stronger than in the other countries. Observations reveal a long-term trend of uninterrupted fall. This drop strongly contrasts with the European context. While nearly all 21 European countries observed have seen a growth in the number of GPs per 100,000 people over the last 20 years with only 2 exceptions, Italy has exhibited a permanent trend of worsening. This trend is even more relevant in the context of the aging population: the European average variation in the over-65 population is 27.9%, and in Italy, it is even higher (29.3%).

Moreover, ISTAT provides data about the number of MMG until 2021. The trend from 2014 up to 2021 is constantly decreasing: in 2014, there were 74.9 MMG over 100.000 in.; in 2021, the data were 68.1, passing from 45,203 doctors to 40,250.

## Discussion

The COVID-19 pandemic has tested the healthcare systems in their capacity to face such relevant shock impacts, focusing on their resilience (Legido-Quigley et al., 2020). GPs have played the frontline of health leaders during the crisis period (Rebnord et al., 2023; Suar et al., 2023; Ares-Blanco et al., 2024), showing GPs services strengths and weaknesses (Greenhalgh et al., 2020; Burau et al., 2024).

This study has analyzed Italian GP policy results from a comparative European perspective in the last two decades using GP number as a proxy of the healthcare policy reform process in primary care. Considering the key role of GPs in healthcare systems (Starfield, 1994; Gulliford, 2002; Starfield et al., 2005; Kringos et al., 2010; Haggerty et al., 2013; Kringos et al., 2013), and despite differences in European healthcare systems (Wendt et al., 2009; Groenewegen et al., 2014), Italy has decreased its number of GPs by almost 10% during the last two decades. This has been an opposite trend compared with most European countries (increasing 6% in the EU average). These reversing healthcare reform trends must also be considered in the context of an increasingly aging population, which is higher in Italy (29.3%) than in the rest of the European countries (27.9%).

Italy had some of the highest incidence of COVID-19 deaths (Statista, 2020). This study suggests that Italy has weakened GP services in Italy from a European comparative perspective and that this might have affected its capacity to manage the pandemic emergency. Nevertheless, further studies will be necessary to investigate the impact of structural data, such as GP numbers, on healthcare system performance (Hogg et al., 2008; Kringos et al., 2013).

This study does not suggest a cause–effect relationship between GP availability and COVID-19 mortality; nonetheless, it proposes that in the European comparative perspective, Italy’s lower investment in GP and primary care in the last decades might have reduced the Italian public healthcare system’s capacity to respond to the COVID-19 pandemic, confirming that in Italy the pandemic has highlighted the unpreparedness of the health system to face the situation; because the reforms adopted over the last 30 years had reduced the public health system capacity (Mauro and Giancotti, 2021). Decades of tight fiscal policy have left the Italian healthcare system more vulnerable in coping with COVID-19 care: the GP policy reform process has left the Italian national healthcare system less equipped than the other EU countries to face the pandemic (Prante et al., 2020; Vicarelli and Giarelli, 2021).

In terms of policy recommendations for Italy, the reform process of the last two decades needs to be put at the center of political debate toward a reform process to increase the role of primary care in Italy as it has been at the moment put on the policy agenda (Vicarelli and Giarelli, 2021). The results of this analysis show the need to reconsider the reform process in Italy and the need to put GPs at the center of health policy reforms even more to manage any health emergency. The recent reforms highlight radical changes in the healthcare policy in Italy toward the introduction of new primary care services offered within the new “Community House” (in Italian: *Casa della Comunità*); nevertheless, its implementation path is presenting several challenges (Genova et al., 2023; Giarelli, 2023; Ingrosso, 2023; Mauro and Giancotti, 2023). The new “Community Houses” are going to be the space in which an innovative vision of primary care and GP roles are supposed to be redefined. This outlines a space of conflict within the GP community and GP unions, as well as in the relationships between GPs and other professionals such as community nurses and social workers. Preparedness for new sanitary dramatic events, such as a new pandemic, would need more in-depth analysis and meta reflexion on the process that has so radically reduced GP in Italy and on the potentiality and weakness in the implementation process of the “Communities Houses” (Genova et al., 2023). Nonetheless, GPs play a crucial role in the frontline of health leaders during the crisis period: GP recruitment and recognition of their key function must be put as a priority in policy agenda all over the world (Suar et al., 2023).

## Data availability statement

The original contributions presented in the study are included in the article/supplementary material, further inquiries can be directed to the corresponding author.

## Author contributions

AG: Conceptualization, Writing – original draft, Writing – review & editing. SL: Data curation, Methodology, Writing – original draft, Writing – review & editing.

## Footnotes

^1^ There is no sufficient GP data about Croatia, Romania, Hungary, Slovakia, Latvia, Denmark, Finland, and Norway.
